# Genome-Wide Scan for Selective Sweeps Reveals Novel Loci Associated with Prolificacy in Iranian Sheep Breeds in Comparison with Highly Prolific Exotic Breed

**DOI:** 10.3390/ani14223245

**Published:** 2024-11-12

**Authors:** Hossein Mohammadi, Amir Hossein Khaltabadi Farahani, Mohammad Hossein Moradi, Hossein Moradi-Shahrbabak, Mohsen Gholizadeh, Abouzar Najafi, Marco Tolone, Enrico D’Alessandro

**Affiliations:** 1 Department of Animal Sciences, Faculty of Agriculture and Natural Resources, Arak University, Arak 38156-8-8349, Iran; amfarahanikh@gmail.com (A.H.K.F.); moradi.hosein@gmail.com (M.H.M.); 2 Department of Animal Science, University College of Agriculture and Natural Resources, University of Tehran, Karaj 31587-11167, Iran; hmoradis@ut.ac.ir; 3 Department of Animal Science and Fisheries, Sari Agricultural Sciences and Natural Resources University (SANRU), Sari 4818166996, Iran; m.gholizadeh@sanru.ac.ir; 4 Departments of Animal and Poultry Science, College of Aburaihan, University of Tehran, Pakdasht 33916-53755, Iran; abozar.najafi@ut.ac.ir; 5 Department of Chemical, Biological, Pharmaceutical and Environmental Sciences, University of Messina, V.le F. Stagno d’Alcontres, 98166 Messina, Italy; marco.tolone@unime.it; 6 Department of Veterinary Sciences, University of Messina, Viale G. Palatucci, 98168 Messina, Italy

**Keywords:** candidate genes, F_ST_, hapFLK, Iranian indigenous sheep breeds, prolificacy, selection signatures

## Abstract

Domestication and selection significantly changed phenotypic traits in modern domestic animals. To identify the genomic regions associated with prolificacy in this study, 837 ewes from three Iranian indigenous sheep breeds, consisting of Baluchi, Lori-Bakhtiari, and Zandi uniparous breeds, and one Greek highly prolific dairy sheep, namely Chios, were genotyped using OvineSNP50K arrays. Statistical tests were then performed using different and complementary methods based on either site frequency (F_ST_) and haplotype (hapFLK) between populations, followed by a pathway analysis of the genes contained in the selected regions. The results revealed that for the top 0.01 percentile of the obtained F_ST_ values, 16 genomic regions on chromosomes 2, 3, 4, 7, 8, 9, 13, 14, 16, 18, 19, and 20, and for hapFLK values, 3 regions located on chromosomes 3, 7, and 13, were under selection. A bioinformatic analysis of these genomic regions showed that these loci overlapped with potential candidate genes associated with prolificacy in sheep including *GNAQ*, *COL5A2*, *COL3A1*, *HECW1*, *FBN1*, *COMMD3*, *RYR1*, *CCL28*, *SERPINA14*, and *HSPA2*. These regions also overlapped with some quantitative trait loci (QTLs) linked to prolificacy traits, milk yield, and body weight. These findings suggest that future research could further link these genomic regions to prolificacy traits in sheep.

## Simple Summary

Prolificacy is a crucial economic trait in sheep, directly influencing their productivity and economic efficiency. Our study aimed to identify single nucleotide polymorphism (SNP) loci associated with sheep prolificacy which could be used in future breeding programs to improve fertility. We obtained candidate genes associated with prolificacy in sheep including *GNAQ*, *COL5A2*, *COL3A1*, *HECW1*, *FBN1*, *COMMD3*, *RYR1*, *CCL28*, *SERPINA14*, and *HSPA2*. Moreover, novel candidate genes associated with fecundity, previously unreported in sheep but detected in other species, were identified consisting of *PAEP* (cow), *LRRTM4*, *SLC9A8* (goat), and *ST3GAL1*, *SNRK* (sow). The pathway analysis revealed that these genes were enriched in pathways related to utero embryonic development and various organ and skeletal development processes. It is noteworthy that we also discovered some novel regions with strong signals of selection that had not been described previously. Overall, this study contributes to the identification of causal mutations, genomic regions, and genes influencing prolificacy, offering insights into the genetic architecture of this trait in sheep.

## 1. Introduction

Litter size is one of the most important reproductive traits in sheep, as well as in other domestic animals, and has always been regarded as a critical index affecting reproductive performance and productivity. Sheep present variable litter sizes within and among breeds, attributable to the natural and artificial selection for higher prolificacy during their long breeding history [1].

Artificial and natural selection has generated the many sheep breeds existing worldwide. During selection, the frequency of certain alleles within the population increases and the surrounding associated chromosomal regions show a decrease in polymorphism due to the hitchhiking effect, a phenomenon known as selective signatures [2]. In order to identify such signatures of selection, different statistical methods have been developed, which are generally categorized into two groups based on whether signatures of selection are investigated within populations (intra-population) or between populations (interpopulation). The methods for between-population studies are based on the fixation index (F_ST_) [3], cross-population extended haplotype homozygosity (XP-EHH) [4], and hapFLK [5]. Since each of these statistical tests identifies specific patterns of selection in the genome, the joint use of different statistical tests has been suggested to increase the power of the detection of signatures of selection along the whole genome [6].

Most of the studies that explored selective footprints in sheep have used the single-site differentiation statistic commonly known as the fixation index, F_ST_, and have led to the identification of a range of candidate genes that contribute to reproduction [7,8], fat deposition [9], immune response, growth, skeletal development, and energy metabolism [10,11]. However, the F_ST_ method does not account for the hierarchical structure of populations and assumes that the subpopulations have derived independently from the same ancestral population [5]. To address this deficiency, the hapFLK method, an extension of the FLK statistic based on linkage disequilibrium (LD) [12], was introduced by [5]. The hapFLK statistic computes the signatures of selection by integrating both the hierarchical structure of populations and information on haplotype frequencies, which enhances the power of the detection of signatures of selection. Application of the hapFLK approach has led to the detection of signatures of selection related to fat deposition in three Chinese sheep breeds with extreme tail types [13].

Wang et al. [14] utilized one uniparous Chinese breed vs. three Kazakhstan multiparous breeds to perform a genome-wide scan for selection signatures and detected *ESR1*, *OXTR*, *MAPK1*, *RYR1*, *PDIA4*, and *CYP19A1* genes as the most plausible genes associated with the litter-size phenotype in sheep. Zhang et al. [15] divided two Chinese indigenous sheep breeds into different groups for comparison and identified 11 genes (*ADCY1*, *POU1F1*, *BMPR2*, *HS6ST1*, *LHCGR*, *DNMT3B*, *GHR*, *LIF*, *PAK1*, *TGFB2*, *GNAQ*) that were associated with litter size. Sánchez-Ramos et al. [1] used selective signature analysis between high and low fecundity populations in Katahdin mutton sheep and found that *CNOT11*, *GLUD1*, *GRID1*, *MAPK8*, and *CCL28* genes might be associated with polytocous traits. Zhong et al. [16] clustered six Chinese indigenous sheep breeds into two subpopulations (high and low fecundity) and identified sixteen strong selective genomic windows containing seven functional genes (*BMPR1B*, *PPM1K*, *RHEB*, *HSPA2*, *PPP1CC*, *HVCN1*, and *CCDC63*) associated with litter size. Yang et al. [17] conducted a selection of signature analysis on prolific Suffolk sheep that were divided into two groups including a multi-lamb group with a 275% lambing rate and single-lamb-group ewes with a lambing rate of 117% and found that *ARHGEF4*, *CATIP*, and *CCDC115* genes may regulate polytocous traits in this sheep breed. All the studies mentioned above divided many sheep breeds into two groups (high and low fertility) to conduct selection signature detection. Therefore, it is hard to ignore the role of different genetic backgrounds.

Sheep production constitutes the most important component of the Iranian livestock industry with a total of approximately 50 million heads (https://www.fao.org/faostat/en/#data/QCL, accessed on 7 October 2024.). Twenty-seven breeds and ecotypes have been documented in Iran. Improving the productivity of sheep plays an important role in improving the quality of life of local people and is one of the important goals of sheep breeding. However, the low fertility of most domesticated sheep is one of the factors limiting the development of sheep breeding in Iran [18].

The Iranian sheep breeds used in this study are among the most common main indigenous sheep breeds reared in various parts of Iran, and were collected from the center breeding station. Baluchi sheep are one of the most famous Iranian indigenous sheep breeds, constituting about 30% of the total sheep population in Iran. This breed has an average litter size at birth of 1.28 ± 0.46 [19]. Baluchi sheep are especially well-adapted to the rather harsh conditions in the arid subtropical region of eastern Iran due to their breed-specific characteristics [20]. The Lori-Bakhtiari sheep are an important heavyweight indigenous breed in the southwestern part of Iran, with a population of more than 1.7 million and an average litter size at birth of 1.17 ± 0.38 [21]. The breed is well known domestically as a major source of meat and exhibits good maternal characteristics. The Zandi sheep are one of the medium-size Iranian indigenous breeds known for their high grazing ability, adaptability to the harsh semi-arid conditions, and resistance to common diseases in the reared regions [22]. The average litter size at birth is 1.10 ± 0.33 [18].

Chios sheep are considered a highly productive and prolific dairy breed mostly reared in semi-intensive or intensive farming conditions, and they have a prolificacy of 4.3 with double and triple births [23]. Chios sheep are an exotic breed originating from the Greek island of Chios that were imported to Iran more than 45 years ago [24].

Screening for fertility genes is helpful for marker-assisted breeding to increase reproductive performance in sheep. Uncovering the genetic mechanisms underlying sheep prolificacy is conducive to enhancing the reproduction of Iranian sheep breeds. In this study, we performed selective signatures analysis based on the differences in litter size between three Iranian indigenous breeds and one Greek Chios breed to identify single nucleotide polymorphism (SNP) loci associated with prolificacy. Our findings will provide a valuable resource for a better understanding of the genetic mechanisms of reproduction and facilitate further genetic improvements in litter size in sheep breeding.

## 2. Materials and Methods

### 2.1. Data and Trait Definition

Phenotypic and pedigree data were collected from three Iranian uniparous sheep breeds including Baluchi, Lori-Bakhtiari, and Zandi. The dataset included 13,408 litter size records from Baluchi sheep at the Abbas-Abad Baluchi Sheep Breeding Station in Mashhad, Iran, with a pedigree file encompassing 22,160 animals (518 sires and 6283 dams). For Lori-Bakhtiari sheep, 7590 litter size records were gathered at the Sholi Breeding Station in Charmahal and Bakhtiari Province, Iran, with a pedigree file comprising 10,645 animals (378 sires and 2805 dams). The Zandi sheep dataset included 7147 litter size records from the Khojir Breeding Station in Tehran Province, Iran, with a pedigree file of 11,832 animals (264 sires and 1975 dams).

The data span the period from 1989 to 2017 and represent individual litter size records of purebred ewes from all three breeds. Total prolificacy (TP), defined as the total number of lambs born to a ewe during her productive lifetime, was the primary trait analyzed. To ensure data quality, animals with fewer than three consecutive parity records were excluded from the analysis. Additionally, individuals from each breed were selected based on minimizing pedigree-based relationships to reduce potential confounding due to genetic relatedness.

Furthermore, the data for a highly prolific Greece dairy sheep, namely the Chios sheep breed, were obtained from the Chios Sheep Breeders’ Cooperative, Macedonia, covering the period from 2005 to 2019. A total of 538 Chios female sheep raised on three farms in Northern Greece were included in the current study.

Based on the total number of lambs born to an ewe during her productive lifetime, the four sheep populations were classified into two groups: a low-yield group (LG, control) and a high-yield group (HG, case) to represent extreme individuals within each breed. For each population, significant differences were observed in LS between the HG and LG (*p*-value < 0.05, Mann–Whitney U test).

To detect positively selected genes for TP, 56 Balushi (TP ≤ 3), 84 Lori-Bakhtiari (TP ≤ 3), and 49 Zandi (TP ≤ 3) were labeled as controls, while 249 Chios (TP ≥ 7) were labeled as cases. In total, three indigenous sheep breed (Baluchi, Lori-Bakhtiari, and Zandi) genomes were used as the reference population, with the Chios genome served as the test population (positive control for high litter size) to identify positive selection signature in these sheep populations.

### 2.2. Animal Genotyping and Quality Control

The blood samples of Iranian sheep breeds were collected from flocks that have recently participated in the registration and recording system of the ABCI (Animal Breeding Centre of Iran). A total of 96, 122, and 96 samples from Baluchi, Lori-Bakhtiari, and Zandi were collected for DNA extraction from whole blood by applying a modified salting out protocol [9], respectively, and genotyping was then performed using the Illumina Ovine SNP50 BeadChip (Illumina, San Diego, CA, USA). In addition, published genotyping data of 538 Chios female sheep samples, based on the Illumina Ovine SNP50 BeadChip, were obtained from the Chios Sheep Breeders’ Cooperative, Macedonia, which are available at https://data.mendeley.com/datasets/nvjn8mvwtg/1, accessed on 25 October 2022.

These data were merged and a total of 45,879 autosomal SNPs were available for further analysis. The dataset was filtered to exclude loci with minor allele frequency (<5%), SNPs with a low call rate (<95%), and animals with more than 5% of missing genotypes. To address the effect of closely related animals, the relatedness was estimated as the proportion of identity-by-descent (IBD) between animal pairs using the *related* option in KING v2.2.4. Following quality control (QC) procedures using PLINK v1.90 [25], a total of 42,160 autosomal SNPs and 837 samples remained in the dataset for genomic-based selective sweep analysis. For the SNPs passing QC, their locations were mapped to the ovine genome assembly OAR_V4.0 using information from the Sheep HapMap dataset (http://www.sheephapmap.org, accessed on 20 November 2015).

### 2.3. Analysis of Population Structure

To identify existing population structures, we conducted a population stratification analysis using Principal Component Analysis (PCA). This analysis was performed to determine how the animals were allocated to their groups based on all markers that passed quality control, and to understand the genetic structure of the studied populations. PCA was performed using the prcomp function in R version 4.2.3 package (http://cran.r-project.org, accessed on 16 March 2023).

### 2.4. Detection of Selective Sweeps

Wright’s Fixation Index (FST)

The unbiased fixation index (Theta) estimator proposed by Weir and Cockerham was calculated to detect signatures of selection [3,26], in R version 4.2.3 (http://www.r-project.org/, accessed on 16 March 2023). The F_ST_ outlier method, which identifies outliers in F_ST_ values among adjacent SNP markers was employed to detect selective sweeps across the genome between low and high prolificacy breeds [27,28]. The value of F_ST_ can theoretically range from 0 to 1, where values close to 0 refer to a low genetic differentiation (i.e., the two populations have no genetic differentiation and share many fixed loci), while values close to 1 indicate significant genetic difference between populations [2,29].

A modified sliding window (SW) approach (termed “Creeping Window”: CW) was used to scan the entire genome for evidence of selective sweeps, with a one SNP step size [30]. For each set of ten adjacent SNPs, the average F_ST_ values was calculated and termed windowed F_ST_. A window size of ten markers was chosen as it appeared to provide the better signal compared with other arbitrary window sizes [31]. The windowed F_ST_ values were then plotted against genome location. All scripts for calculating Wright (F_ST_) and Weir and Cockerham fixation indices were written and performed in R v4.2.3. The top 0.01 of F_ST_ windows were then used to discover selection signatures, following Kijas et al. [32] and Moradi et al. [9].

#### Selection Signature Detection Using hapFLK

In this study, hapFLK was also utilized to detect the selection signatures in two sheep population groups with obviously different litter sizes. The hapFLK method, proposed by Fariello et al. [5], is a haplotype-based approach that can be applied to unphased genotypic data. This method relies on differences in haplotype frequencies between populations, with haplotype information being estimated using fastPHASE 1.4. No outgroups were defined in this study, and 10 clusters (−K 10) were used for the fastPHASE model. The hapFLK statistic was computed for 20 expectation-maximization (EM) runs to fit the linkage disequilibrium (LD) model (−nfit = 20). The hapFLK 1.4.0 program is available at https://forge-dga.jouy.inra.fr/projects/hapflk/files, accessed on 2 February 2022.

Since hapFLK values for each SNP across the entire genome approximately follow the normal distribution, the hapFLK values were further standardized to calculate the *p*-values. The formula is as follows:

$${\mathrm{hapFLK}}_{\mathrm{adj}}=\frac{\mathrm{raw hapFLK}-\mathrm{mean (raw hapFLK)}}{\mathrm{SD(raw hapFLK)}}$$

where mean and SD (raw hapFLK) are average and standard deviation of raw hapFLK, respectively. Thus, the standardized hapFLK (Z scores) roughly follows a standardized normal distribution. Subsequently, the *p*-values were estimated based on a chi-square distribution, using the hapFLK values generated for each SNP, as described by Fariello et al. [5]. Similar to the F_ST_ test, candidate selection SNPs were defined as those ranking in the top 0.01 percentile of the hapFLK values.

### 2.5. Identification of Potential Candidate Genes and QTLs and Pathway Analysis

Genes located in the genomic regions significantly differentiated between low and high prolificacy breeds were acquired using the data mining tool BioMart (http://asia.ensembl.org/biomart/martview, accessed on 26 June 2024.), with the reference assembly of the *O. aries* genome OAR v4.0. Nearby genes within a flanking distance of 200 kb from each region were acquired (https://www.ncbi.nlm.nih.gov/assembly/GCF_000298735.2, retrieved on 15 June 2022). This distance was previously selected by Moradi et al. [9] for sheep and by Zhou et al. [33] for cattle. The protein-coding genes which overlapped with the regions under positive selection and have previously been confirmed to be associated with prolificacy were considered as potential candidate genes. To determine the biological functions of each gene, the Kyoto Encyclopedia of Genes and Genomes (KEGG) pathway [34] and Gene Ontology (GO) for biological processes [35] for all identified genes in candidate regions were studied using DAVID annotation, DAVID v6. [36]. In addition, in order to examine the overlap of the putative genomic regions under selection with previously known QTLs, cattle QTL database (*UMD-3.1*) was used because the sheep genome has not been properly annotated. Finally, a comprehensive literature review was conducted to verify whether these genes have some relevance with litter size in sheep or other mammals.

## 3. Results

### 3.1. Population Genetic Structure

**Figure 1:**
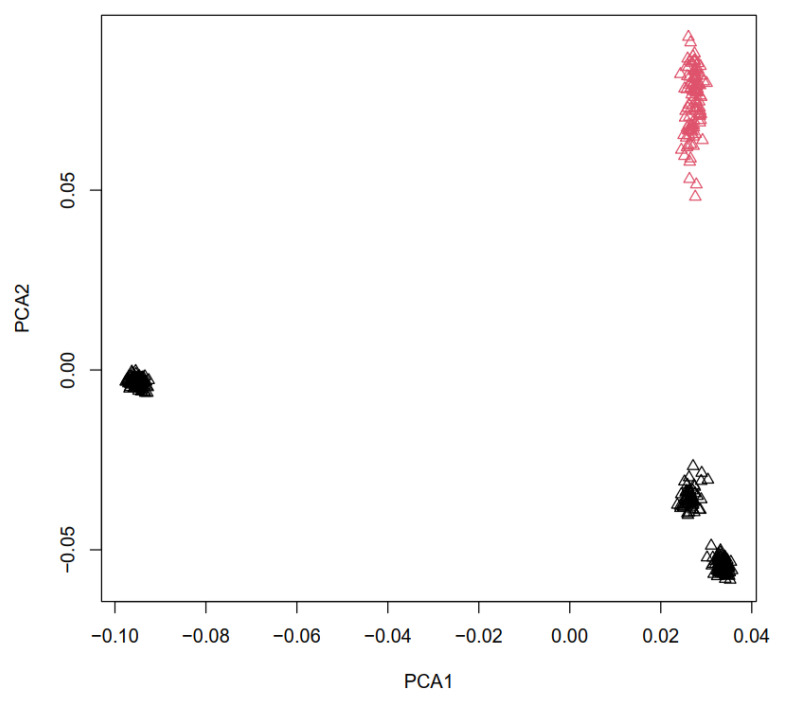
Genetic relationship defined with Principal Component Analysis between three Iranian sheep breeds (black) and Chios sheep breed (red). Principal components diagram provided based on the genomic kinship coefficients between individuals. The first two principal components (PCA1 and PCA2) and the variance explained by each component are shown on the corresponding axis.

Figure 1 shows the results of population genetics analysis among four sheep populations. We performed PCA to identify how animals allocated to their true population in this study. The results clustered four distinct populations according to geographical origins and type breed (Figure 1). The PCA results demonstrated that all the animals were accurately assigned to their respective original groups. The Lori-Bakhtiari, Baluchi, and Zandi breeds were distinctly separated along PC1, whereas the Chios breed was differentiated along PC2 (Figure 1). The first and second PCs (PC1 and PC2) accounted for 22.98 and 17.48% of the total variation, respectively.

In the PCA analysis conducted, it was observed that closely related breeds clustered together, with the Lori-Bakhtiari, Baluchi, and Zandi breeds serving as prime examples. Two differentiated genetic groups were observed from the animal population, which additionally agree with the phenotypical classification given by the prolificacy. On the other hand, Chios sheep were clearly differentiated from all other breeds.

### 3.2. Identifying the Genomic Regions with Selection Signals Using F_ST_

**Figure 2:**
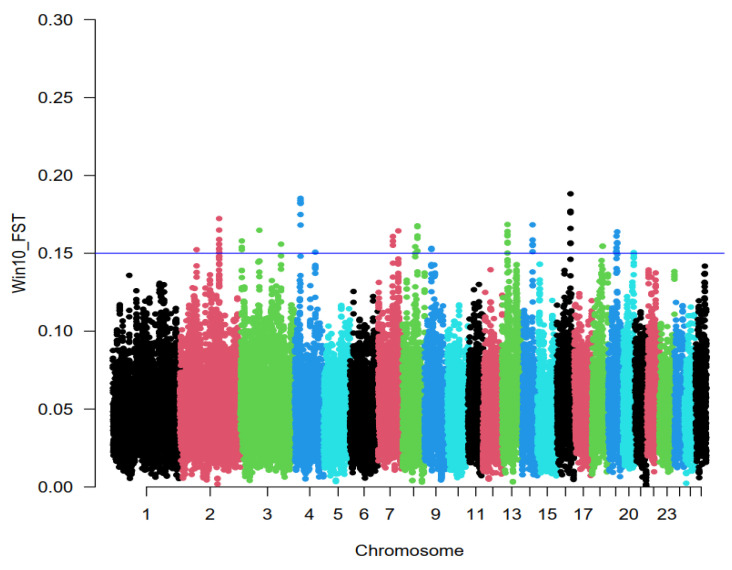
Genome-wide distribution of pairwise unbiased F_ST_ (Theta) between three Iranian indigenous (low litter size) and exotic Chios (high litter size) breeds. Overlapping windows of 600 kb across the genome were used to identify putative signatures of selection. The threshold determined based on the 0.01% of the empirical genome-wide distribution is shown by the blue line. The SNP position on different chromosomes shown on the X-axis, and Win10 F_ST_ values are plotted on the Y-axis.

Averaged Weir and Cockerham’s unbiased F_ST_ values obtained for sliding windows of 600 kb across the genome gave an unbiased average of between-population F_ST_ of 0.0517 (SD = 0.023), as shown in Figure 2. Evidence of selection was found in 16 regions which contained 0.01% of windows with the highest F_ST_ values over 0.15 included in 42 significant windows. These regions were located on chromosomes 2 (58,551–59,151 and 119,989–1,120,589 kb), 3 (3456–4056, 47,927–48,527, and 166,043–166,643 kb), 4 (22,288–22,888, and 78,819–79,419 kb), 7 (59,416–60,016 kb), 8 (60,397–60,997 kb), 9 (20,526–21,126 kb), 13 (22,489–23,089 kb), 14 (47,470–48,070 kb), 16 (31,025–31,625 kb), 18 (57,744–58,344 kb), 19 (14,930–15,530 kb), and 20 (44,180–44,780 kb).

### 3.3. Identifying the Genomic Regions with Selection Signals Using hapFLK

**Figure 3:**
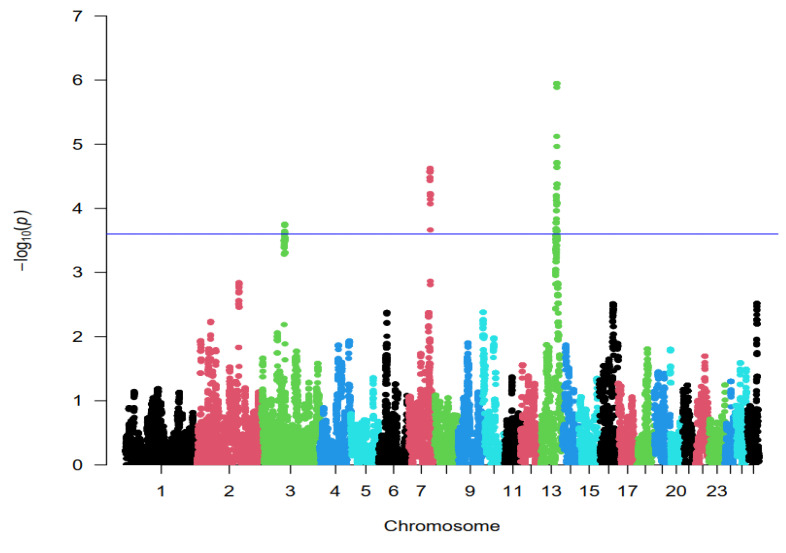
Genome-wide distribution of selection signatures detected by hapFLK analysis between three Iranian indigenous (low litter size) and exotic Chios (high litter size) breeds, with a blue line representing a cutoff threshold of 0.01 percentile. The SNP position on different chromosomes shown on the X-axis, and hapFLK values are plotted on the Y-axis.

The hapFLK statistic was implemented to find genomic regions under selection. As shown in Figure 3, the distribution of hapFLK values approximately follows the normal distribution. *p*-values for hapFLK calculated using an empirical method is appropriate because of ascertainment bias in the SNP panel [5]. Therefore, the *p*-value of each SNP was obtained from a normal distribution. Negative log_10_ *p*-values plotted in genomic order revealed three regions under strong selection. The hapFLK analyses detected regions of putative selection signature regions, notably with strong signals at chromosomes 3 (47,927–48,527 kb), 7 (74,411–75,011 kb), and 13 (77,582–78,182 kb) (Figure 3 and Table 1). Only the genomic region in chromosome 3 was detected by both F_ST_ and hapFLK methods.

### 3.4. Candidate Genes

According to the information from the Ovis_aries_v4.0 genome, a total of 13 candidate genes associated with prolificacy were obtained, through the F_ST_ method (Table 1). On the other hand, with the hapFLK method, two other candidate genes associated with prolificacy were found (Table 1), different from those detected by the F_ST_ method. Based on GO annotation and KEGG enrichment analysis, 15 genes (*GNAQ, COL5A2, COL3A1, HECW1, FBN1, COMMD3, RYR1, CCL28, SERPINA14, HSPA2*, *PAEP, LRRTM4, ST3GAL1, SNRK,* and *SLC9A8*) were significantly associated with litter size. Once these genes were selected through selection signatures, they were filtered according to functions previously reported as being associated with reproduction in sheep, goat, cattle, and pigs.

### 3.5. QTL Overlapping with Signatures of Selection

Quantitative trait loci associated with litter size that overlapped with detected signatures of selection were retrieved from the cattle QTL databases. Analysis of the overlaps between the signatures of selection and reported QTL indicated that most of the detected regions contained a QTL (Table 1). Some QTL for traits of reproductive traits, such as fertility index, calving ease, scrotal circumference, and interval to first estrus after calving overlapped significantly with putative regions of the signatures of selection. We also found significant overlaps of QTL related to morphology, milk production, and growth traits (such as udder depth, udder height, milk yield, average daily gain, and body weight) with putative regions.

### 3.6. Gene Ontology Enrichment Analysis

For the gene sets found in the detected genomic regions of the two methods according to the top 0.01 percentile, the GO terms including biological processes, cellular component, molecular functions, and KEGG pathways were surveyed using the DAVID database. Through the functional annotation analyses, genes involved in reproduction-related processes or pathways were analyzed together to find candidate genes related to prolificacy.

GO analysis revealed that these genes were significantly enriched in eight GO terms (*p* < 0.05), including in utero embryonic development, embryo development ending in birth or egg hatching, embryonic skeletal system morphogenesis, calcium channel complex, etc. Three candidate genes: *SNRK*, *HSPA2*, and *RYR1* were identified by both F_ST_ and hapFLK (Table 2), and another five genes were detected by F_ST_. All these genes were involved in GO terms or pathways.

## 4. Discussion

As litter size is an important limiting factor affecting the reproductive efficiency of sheep, the identification and use of molecular markers to increase litter size can effectively accelerate the development of the sheep-breeding industry. Zhong et al. [16] divided six Chinese sheep breeds into two groups with different lambing performances according to the litter size in the first parity. Genome wide signature was analyzed using the F_ST_ method, and a total of 71 genome-wide selection regions and *RHEB*, *HSPA2*, *PPP1CC*, *HVCN1*, and *CCDC63* candidate genes related to litter size were identified. In the study of Sánchez-Ramos et al. [1], a genome-wide selection signatures analysis of two groups of 48 Katahdin ewes with different lambing performances revealed eleven new candidate genes, including *CNOT11*, *GLUD1*, *GRID1*, *MAPK8*, *CCL28*, *ANK2*, *ARHGAP22*, *GHITM*, *HERC6*, *DPF2*, and *TRNAC-GCA* related to litter size. Based on these findings, this study selected the total number of lambs in multiple parities for analysis, hoping to improve the accuracy of previous studies.

To determine whether Iranian and Chios sheep populations were suitable for the selection signature analysis, we conducted a population structure analysis. In genome-wide signature-of-selection studies, the use of multivariate analysis is common to characterize the genetic structure of the populations. The most used method is the PCA [17,37,38,39]. As in other studies with sheep [7,17,38], in this study, the PCA made it possible to graphically observe the genetic differences of four sheep populations.

Detecting recent positive selection signatures in domesticated animals that have gone through both artificial and natural selection can provide information on genomic sites that can contribute to the identification of beneficial mutations and underlying biological pathways for economically important traits. The whole-genome single locus F_ST_ statistics (population differentiation-base) and haplotype-based hapFLK (linkage disequilibrium-base) were calculated for comprising one multiparous breed (Chios) and the three uniparous breeds (Baluchi, Lori-Bakhtiari, and Zandi).

Among multiple populations, common selection signatures can be detected using the fixation index (F_ST_). However, hidden population substructures exist in multiple breeds, which cannot be considered by F_ST_, due to its simplicity, and can lead to false positives. Moreover, these substructures are common in domestic animal populations. To address this limitation, a haplotype-based approach (hapFLK) was proposed that accommodates population substructures [5]. The hapFLK was implemented to detect selection signatures through haplotype differentiation among hierarchically structured populations.

Hence, hapFLK can integrate the information from differences in haplotype allele frequencies among populations, the local haplotype data, and population substructures to identify genomic regions showing under selection. Thus, hapFLK can identify a recent positive selection that results in a beneficial mutation residing on a haplotype with a higher frequency than average and a longer length. However, only one putative region on chromosome 3 (47,927–48,527 kb) intersected between F_ST_ and hapFLK. The lack of overlap for the top putative regions is probably due to hapFLK being designed to mostly detect regions with migration and bottlenecks [5]. In addition, the identification of true signatures is complicated and of great concern because of false positives arising from selection signatures [40]. Estimating the degree of such bias on selection signature analyses, fewer efforts have been applied to date. In this study, a stringent percentile was applied for all the approaches to limit possible false positives.

In the present study, we identified ten important candidate genes of litter size in sheep (*GNAQ*, *COL5A2*, *COL3A1*, *HECW1*, *FBN1*, *COMMD3*, *RYR1*, *CCL28*, *SERPINA14*, *HSPA2*). Moreover, another five genes (*PAEP*, *LRRTM4*, *ST3GAL1*, *SNRK*, and *SLC9A8*) have been associated with reproductive traits in other species, which indicate that these five genes play important role in litter size in sheep breeds. These findings indicate that these fifteen genes may be important candidate genes related to fecundity performance. Their functional verification needs further investigation and validation using full genome sequences and expression studies in the future.

*GNAQ* is located on chromosome 2 in sheep. Previous studies have shown that the *GNAQ* gene is significantly associated with small size in different sheep breeds [15,41]. Our study adds to this point that the *GNAQ* gene was also significantly associated with small size in a comparison of the two groups.

*COL5A2* and *COL3A1* are located on chromosome 2. The collagen-type genes *COL3A1* and *COL5A2* are only 40 kb apart, and they contribute to osteogenesis, normal bone formation, skeletal development, myogenesis, and muscle growth [42], also resembling the functionality of the major genes. Tsartsianidou et al. [23] used genome-wide association analysis to study reproductive traits in prolific Chios sheep and found *COL5A2* and *COL3A1* were significantly associated with the age-first lambing trait, and this gene could also serve as a candidate gene affecting reproductive traits in sheep.

*PAEP* is located on chromosome 3 in sheep. *PAEP* is a progesterone-associated endometrial protein, and studies demonstrate that this gene enhances cell proliferation through the receptor-mediated membrane IgM receptor, and is the main component in regulating cell proliferation in milk [43]. As a 28-kD glycoprotein, *PAEP* constitutively expresses in both the human reproductive tract and the hematopoietic system. The gene *PAEP* was reported as the candidate gene for pregnancy rates in Chinese Holstein Cattle [44]. Therefore, *PAEP* promotes cell proliferation under the action of progesterone, which has a positive impact on the pregnancy matching rate in adult sheep.

The *LRRTM4* gene, a candidate gene for goat high fecundity revealed by selection signature analysis, is located on chromosome 3 in sheep and is significantly associated with prolificacy in sheep. According to our findings, the *LRRTM4* gene, which was identified by both Fst and hapFLK methods, could be the gene with the most potential to affect sheep litter size. Sun et al. [45] explored the genetic variation of *LRRTM4* in goat and evaluated the genetic impact on litter size. Genome-wide analysis in Brazilian cattle breeds showed that this gene is involved in biological processes related to fertility [46]. By UniProt comparison, the *LRRTM4* protein sequence of sheeps and goats are as high and the *LRRTM4* gene, as a candidate gene for the prolificacy of goats, may have some influence on the reproductive traits of sheep population.

*HECW1* (HECT, C2, and WW domain-containing E3 ubiquitin protein ligase 1) is located on ovine chromosome 4. Previous studies have shown that the *HECW1* gene could be regarded as a positional and functional candidate gene for reproductive traits in sheep. *HECW1* has been reported to be significantly associated with total lambs born in the Luzhong mutton sheep population of China [37].

*FBN1* is located on ovine chromosome 7. As the main component of microfibrils in the extracellular matrix, the gene *FBN1* regulates cumulus cell apoptosis by reducing the expression level of *BMP15* involved in estrogen signaling in porcine ovaries [47]. *FBN1* has been identified by GWAS as a candidate gene associated with litter size in high prolific Wadi sheep [48].

*ST3GAL1* is located on chromosome 9 in sheep. *ST3GAL1* is a key sialyltransferase which adds α2,3-linked sialic acid to substrates and generates core 1 *O*-glycan structure. *SNRK*, located on chromosome 19, is a SNF1-related kinase with a considerable expression level in the testis. It is activated by liver kinase B1 (LKB1) and in this context is suggested to play a role in sperm release from the germinal epithelium [49]. These two genes have been reported as candidates for reproductive traits such as semen quality and litter weight at weaning in different pig populations [15,50] and require experimental validation in sheep breeds.

*COMMD3* is located on ovine chromosome 13. The COMMD family member COMM domain containing 3 (COMMD3) has been recently identified as a regulator of human epidermal growth factor receptor 2 (HER2) endosomal trafficking [51]. *COMMD3*, associated with longevity, has been related to length of time in the flock in U.S. rangeland ewes [52].

*RYR1* (ryanodine receptor) is located on ovine chromosome 14. *RYR1* was identified as regulating calcium release in oocytes, which also play roles in oocyte maturation [53]. In a selection signature analysis of reproductive traits in Chinese and Kazakhstan sheep breeds, the *RYR1* gene was identified as being involved in Oxytocin-signaling mechanisms and acting on pituitary gonadotropin secretion [14]. This is consistent with our findings.

*CCL28* is located on ovine chromosome 16. The *CCL28* (C-C motif chemokine ligands 28) was found to have a high expression during early fetal development in sheep [54]. In particular, the *CCL28* gene is associated with the recruitment of immune cells in the spleen and the nasal mucosa of the fetus, which leads to the development of a functional immunological system pre-birth. In Katahdin ewes, the *CCL28* gene was reported as a candidate for the number of lambs born per ewe trait [1].

*SERPINA14* is located on ovine chromosome 18. Uterine serpin (*SERPINA14*) plays crucial roles in the pregnancy of farm animals, such as nutrient supply to the conceptus, immunoregulation, and maternal recognition of pregnancy [55]. *SERPINA14* was identified as being associated with the follicular phase in sheep breeds [37].

*HSPA2* is located on ovine chromosome 7. *HSPA2* was identified as participating in the estrogen-signaling pathway and thyroid hormone-signaling pathway. These pathways played a crucial role in the secretion and regulation of hormones. This significant finding is consistent with the candidate gene obtained by Zhong et al. [16], using comparative genomics using selection signature analysis methods for litter size with different lambing performances.

*SLC9A8* is located on ovine chromosome 13. Some studies have found that *SLC9A8* is one of the regulatory genes of the sodium/proton reverse transporter (NHE) protein. The NHE protein is a membrane protein found in most organisms, where mice without NHE8 will have no acrosome, abnormal mitochondrial distribution, and decreased motor ability, resulting in infertility [55]. Fang et al. [56] conducted a genome-wide association study on Dazu black goats, a prominent Chinese local goat breeds renowned for its exceptional reproductive performance. Their study identified SLC9A8 as a gene associated with average litter size in these goats.

In addition, the GO categories as well as KEGG pathways showed that these genes played an essential role in embryo growth and the ovulation of ewes. However, further expression analyses of these genes in Baluchi, Lori-Bakhtiari, and Zandi sheep breeds are necessary in future studies. Taken together, the apparent differences in litter size among the breeds might be explained by diverse regulation mechanisms.

## 5. Conclusions

The grouping of breeds successfully identified general genomic regions selected for litter size. This study is the first to identify candidate genes for fecundity in different Iranian sheep breeds when compared with the exotic Chios breed. These candidate genes were detected through the selection signatures FST and hapFLK, which confirmed the influence of the ten genes *GNAQ*, *COL5A2*, *COL3A1*, *HECW1*, *FBN1*, *COMMD3*, *RYR1*, *CCL28*, *SERPINA14*, and *HSPA2* on the characteristics of fertility in sheep; in addition, five other new candidate genes were detected that had not been previously reported in sheep: *PAEP* (cow), *LRRTM4*, *SLC9A8* (goat), and *ST3GAL1*, *SNRK* (sow). Our results provide novel insights into the genetic architecture of sheep reproductive traits, focusing on the total prolificacy. These genomic regions could serve as a starting point for identifying gene mutations and to select for high prolific animals.

## Figures and Tables

**Table 1:** Selective sweeps and candidate genes putatively selected by two statistical methods of selection signature detection affecting litter size in sheep and reproduction traits reported in other studies.

Chr	Location	Genes	QTL	QTL-ID	Method
2	58851035	*PSAT1, CEP78, CEP78, GNAQ, GNA14*	Milk yield	22449276	F_ST_
2	120289850	*SLC40A1, WDR75, COL5A2, COL3A1*	Milk yield	25148050	F_ST_
3	3756078	*NACC2, UBAC1, CAMSAP1, KCNT1, SOHLH1 GLT6D1, PAEP, GBGT1, RALGDS, CEL, GTF3C5, GFI1B, TSC1, SPACA9, AK8, GTF3C4, DDX31*	-	-	F_ST_
3	48227408	*CCT4, FAM161A, XPO1, USP34, AHSA2, LRRTM4, COMMD1*	Milk Yield (180d)	32510640	F_ST_, hapFLK
3	166343845	*HAL, LTA4H, ELK3, CDK17, CFAP54, NEDD1*	Fertility index	26369327	F_ST_
4	22588784	*ETV1*	-	-	F_ST_
4	79119938	*NUDCD3, CAMK2B, YKT6, GCK, MYL7, POLD2, AEBP1, POLM, BLVRA, STK17A, HECW1*	Average daily gain	23851991	F_ST_
7	59716677	*COPS2, SECISBP2L, SHC4, CEP152, FBN1, DUT, SNORA72*	Calving ease	22888914	F_ST_
8	60697777	*ALDH8A1, HBS1L, MYB, AHI1, PDE7B*	Body weight gain	19966163	F_ST_
9	20826481	*ZFAT, ST3GAL1, NDRG1, CCN4, TG2*	Scrotal circumference	28852499	F_ST_
13	22789644	*MLLT10, DNAJC1, COMMD3, BMI1, SPAG6, PIP4K2A*	Udder height	26883850	F_ST_
14	47770629	*SIPA1L3, DPF1, PPP1R14A, SPINT2, C14H19orf33, YIF1B, KCNK6, CATSPERG, PSMD8, GGN, SPRED3, FAM98C, RASGRP4, RYR1, MAP4K1, EIF3K, ACTN4, LGALS4, ECH1, HNRNPL, RINL, SIRT2, NFKBIB, CCER2 SARS2, MRPS12, FBXO17, ACP7, PAK4*	Interval to first estrus after calving	28814769	F_ST_
16	31325831	*NNT, PAIP1, C5orf34, TMEM267, CCL28, HMGCS1, NIM1K, ZNF131*	Milk yield	27006194	F_ST_
18	58044513	*DICER1, CLMN, SERPINA14, SYNE3, SCARNA13, TUNAR*	Udder depth	21831322	F_ST_
19	15230063	*NKTR, ZBTB47, KLHL40, HHATL, CCDC13, HIGD1A, CYP8B1, GASK1A, POMGNT2, SNRK, ANO10, SNORD36, ABHD5*	Milk yield (daughter deviation)	22449276	F_ST_
20	44480429	*APPL1, HESX1, IL17RD, ARHGEF3, TASOR CCDC66, ERC2*	-	-	F_ST_
7	74711971	*ESR2, MTHFD1, AKAP5, ZBTB25, ZBTB1, HSPA2, PPP1R36, SPTB, CHURC1, GPX2, RAB15, FNTB, PLEKHG3*	Body weight	26272623	hapFLK
13	77882134	*KCNB1, PTGIS, B4GALT5, SLC9A8, SPATA2 RNF114, SNORA70, SNAI1, CEBPB*	Average daily gain	23038745	hapFLK

**Table 2:** The candidate genes for litter size under selection by two methods of analysis from the critical KEGG pathways and GO terms.

Candidate Genes	Term	GO-ID	Count	*p*-Value	Test
*COMMD3, COL3A1*	in utero embryonic development	0001701	5	0.0079	F_ST_
*COL3A1*	muscle tissue development	0060537	4	0.037	F_ST_
*COL5A2,* *COMMD3, COL3A1*	skeletal system development	0001501	5	0.038	F_ST_
*COMMD3, COL3A1*	embryo development ending in birth or egg hatching	0009792	5	0.045	F_ST_
*SNRK, HSPA2*	microtubule cytoskeleton	0015630	12	0.024	F_ST_. hapFLK
*HSPA2, RYR1*	calcium channel complex	0034704	3	0.043	F_ST_. hapFLK
*GNAQ*	enzyme regulator activity	0030234	14	0.020	F_ST_
*ST3GAL1*	metabolic pathways	oas01100	23	0.0060	F_ST_

## Data Availability

All data including genotypic data and phenotypes used in the current study that support the findings of this study are available from the corresponding author upon reasonable request. The phenotypes used in the current study were under legal restrictions and commercial ownership set by the Iran National Animal Breeding Center and Promotion of Animal Products and can be made available for academic purposes after signing a Material Transfer Agreement. Also, data for Chios sheep avialiable at https://data.mendeley.com/datasets/nvjn8mvwtg/1, accessed on 25 October 2022.

## References

[1] Sánchez-Ramos R., Trujano-Chavez M.Z., Gallegos-Sánchez J., Becerril-Pérez C.M., Cadena-Villegas S., Cortez-Romero C. (2023). Detection of Candidate Genes Associated with Fecundity through Genome-Wide Selection Signatures of Katahdin Ewes. Animals.

[2] Moradi M.H., Nejati-Javaremi A., Moradi-Shahrbabak M., Dodds K.G., Brauning R., McEwan J.C. (2022). Hitchhiking Mapping of Candidate Regions Associated with Fat Deposition in Iranian Thin and Fat Tail Sheep Breeds Suggests New Insights into Molecular Aspects of Fat Tail Selection. Animals.

[3] Weir B.S., Hill W.G. (2002). Estimating F-Statistics. Annu. Rev. Genet..

[4] Sabeti P.C., Varilly P., Fry B., Lohmueller J., Hostetter E., Cotsapas C., Xie X., Byrne E.H., McCarroll S.A., The International HapMap Consortium (2007). Genome-wide detection and characterization of positive selection in human populations. Nature.

[5] Fariello M.I., Boitard S., Naya H., SanCristobal M., Servin B. (2013). Detecting Signatures of Selection Through Haplotype Differentiation Among Hierarchically Structured Populations. Genetics.

[6] Rostamzadeh Mahdabi E., Esmailizadeh A., Ayatollahi Mehrgardi A., Asadi Fozi M. (2021). A genome-wide scan to identify signatures of selection in two Iranian indigenous chicken ecotypes. Genet. Sel. Evol..

[7] Tao L., He X., Jiang Y., Liu Y., Ouyang Y., Shen Y., Hong Q., Chu M. (2021). Genome-Wide Analyses Reveal Genetic Convergence of Prolificacy between Goats and Sheep. Genes.

[8] Tian D., Han B., Pei Q., Zhou B., Wang L., Li X., Zhao K. (2023). Whole genome sequencing identified candidate genes related to litter size of Qinghai fine wool sheep under artificial selection. Small Rumin. Res..

[9] Moradi M.H., Nejati-Javaremi A., Moradi-Shahrbabak M., Dodds K.G., McEwan J.C. (2012). Genomic scan of selective sweeps in thin and fat tail sheep breeds for identifying of candidate regions associated with fat deposition. BMC Genet..

[10] Yurchenko A.A., Deniskova T.E., Yudin N.S., Dotsev A.V., Khamiruev T.N., Selionova M.I., Egorov S.V., Reyer H., Wimmers K., Brem G. (2019). High-density genotyping reveals signatures of selection related to acclimation and economically important traits in 15 local sheep breeds from Russia. BMC Genom..

[11] Liu Z., Tan X., Wang J., Jin Q., Meng X., Cai Z., Cui X., Wang K. (2022). Whole genome sequencing of Luxi Black Head sheep for screening selection signatures associated with important traits. Anim. Biosci..

[12] Bonhomme M., Chevalet C., Servin B., Boitard S., Abdallah J., Blott S., SanCristobal M. (2010). Detecting Selection in Population Trees: The Lewontin and Krakauer Test Extended. Genetics.

[13] Zhao F., Deng T., Shi L., Wang W., Zhang Q., Du L., Wang L. (2020). Genomic Scan for Selection Signature Reveals Fat Deposition in Chinese Indigenous Sheep with Extreme Tail Types. Animals.

[14] Wang Y., Niu Z., Zeng Z., Jiang Y., Jiang Y., Ding Y., Tang S., Shi H., Ding X. (2020). Using High-Density SNP Array to Reveal Selection Signatures Related to Prolificacy in Chinese and Kazakhstan Sheep Breeds. Animals.

[15] Zhang Z., Sui Z., Zhang J., Li Q., Zhang Y., Wang C., Li X., Xing F. (2022). Identification of Signatures of Selection for Litter Size and Pubertal Initiation in Two Sheep Populations. Animals.

[16] Zhong T., Hou D., Zhao Q., Zhan S., Wang L., Li L., Zhang H., Zhao W., Yang S., Niu L. (2024). Comparative whole-genome resequencing to uncover selection signatures linked to litter size in Hu Sheep and five other breeds. BMC Genom..

[17] Yang H., Zhu M., Wang M., Zhou H., Zheng J., Qiu L., Fan W., Yang J., Yu Q., Yang Y. (2024). Genome-wide comparative analysis reveals selection signatures for reproduction traits in prolific Suffolk sheep. Front. Genet..

[18] Mohammadi H., Moradi Shahrebabak M., Moradi Shahrebabak H., Vatankhah M. (2012). Estimation of genetic parameters of reproductive traits in Zandi sheep using linear and threshold models. Czech J. Anim. Sci..

[19] Yadollahi S., Gholizadeh M., Hafezian H. (2019). Bayesian inference on genetic parameters for some reproductive traits in sheep using linear and threshold models. Small Rumin. Res..

[20] Gholizadeh M., Rahimi-Mianji G., Nejati-Javaremi A., De Koning D.J., Jonas E. (2014). Genomewide association study to detect QTL for twinning rate in Baluchi sheep. J. Genet..

[21] Abdoli R., Mirhoseini S.Z., Ghavi Hossein-Zadeh N., Zamani P., Ferdosi M.H., Gondro C. (2019). Genome-wide association study of four composite reproductive traits in Iranian fat-tailed sheep. Reprod. Fertil. Dev..

[22] Bohlouli M., Mohammadi H., Alijani S. (2013). Genetic evaluation and genetic trend of growth traits of Zandi sheep in semi-arid Iran using random regression models. Small Rumin. Res..

[23] Tsartsianidou V., Pavlidis A., Tosiou E., Arsenos G., Banos G., Triantafyllidis A. (2023). Novel genomic markers and genes related to reproduction in prolific Chios dairy sheep: A genome-wide association study. Animal.

[24] Moosanezhad Khabisi M., Asadi Foozi M., Lv F.-H., Esmailizadeh A. (2021). Genome-wide DNA arrays profiling unravels the genetic structure of Iranian sheep and pattern of admixture with worldwide coarse-wool sheep breeds. Genomics.

[25] Chang C.C., Chow C.C., Tellier L.C., Vattikuti S., Purcell S.M., Lee J.J. (2015). Second-generation PLINK: Rising to the challenge of larger and richer datasets. GigaScience.

[26] Weir B.S., Cockerham C.C. (1984). Estimating *f*-statistics for the analysis of population structure. Evolution.

[27] Akey J.M., Zhang G., Zhang K., Jin L., Shriver M.D. (2002). Interrogating a High-Density SNP Map for Signatures of Natural Selection. Genome Res..

[28] Karlsson S., Moen T. (2010). The power to detect artificial selection acting on single loci in recently domesticated species. BMC Res. Notes.

[29] Barreiro L.B., Laval G., Quach H., Patin E., Quintana-Murci L. (2008). Natural selection has driven population differentiation in modern humans. Nat. Genet..

[30] Qanbari S., Strom T.M., Haberer G., Weigend S., Gheyas A.A., Turner F., Burt D.W., Preisinger R., Gianola D., Simianer H. (2012). A High Resolution Genome-Wide Scan for Significant Selective Sweeps: An Application to Pooled Sequence Data in Laying Chickens. PLoS ONE.

[31] Mokhber M., Moradi-Shahrbabak M., Sadeghi M., Moradi-Shahrbabak H., Stella A., Nicolzzi E., Rahmaninia J., Williams J.L. (2018). A genome-wide scan for signatures of selection in Azeri and Khuzestani buffalo breeds. BMC Genom..

[32] Kijas J.W., Lenstra J.A., Hayes B., Boitard S., Porto Neto L.R., San Cristobal M., Servin B., McCulloch R., Whan V., Gietzen K. (2012). Genome-Wide Analysis of the World’s Sheep Breeds Reveals High Levels of Historic Mixture and Strong Recent Selection. PLoS Biol..

[33] Zhou Y., Utsunomiya Y.T., Xu L., Hay E.H.A., Bickhart D.M., Alexandre P.A., Rosen B.D., Schroeder S.G., Carvalheiro R., Neves H.H.d.R. (2016). Genome-wide CNV analysis reveals variants associated with growth traits in Bos indicus. BMC Genom..

[34] Kanehisa M., Goto S., Sato Y., Furumichi M., Tanabe M. (2012). KEGG for integration and interpretation of large-scale molecular data sets. Nucleic Acids Res..

[35] Ashburner M., Ball C.A., Blake J.A., Botstein D., Butler H., Cherry J.M., Davis A.P., Dolinski K., Dwight S.S., Eppig J.T. (2000). Gene Ontology: Tool for the unification of biology. Nat. Genet..

[36] Huang D.W., Sherman B.T., Lempicki R.A. (2009). Systematic and integrative analysis of large gene lists using DAVID bioinformatics resources. Nat. Protoc..

[37] Tao L., He X.Y., Wang F.Y., Pan L.X., Wang X.Y., Gan S.Q., Di R., Chu M.X. (2021). Identification of genes associated with litter size combining genomic approaches in Luzhong mutton sheep. Anim. Genet..

[38] Paim T.D.P., Alves Dos Santos C., Faria D.A.D., Paiva S.R., McManus C. (2022). Genomic selection signatures in Brazilian sheep breeds reared in a tropical environment. Livest. Sci..

[39] Barani S., Nejati-Javaremi A., Moradi M.H., Moradi-Sharbabak M., Gholizadeh M., Esfandyari H. (2023). Genome-wide study of linkage disequilibrium, population structure, and inbreeding in Iranian indigenous sheep breeds. PLoS ONE.

[40] Gouveia J.J.D.S., Silva M.V.G.B.D., Paiva S.R., Oliveira S.M.P.D. (2014). Identification of selection signatures in livestock species. Genet. Mol. Biol..

[41] Zhu M., Zhang H., Yang H., Zhao Z., Blair H.T., Zhai M., Yu Q., Wu P., Fang C., Xie M. (2022). Polymorphisms and association of *GRM1*, *GNAQ* and *HCRTR1* genes with seasonal reproduction and litter size in three sheep breeds. Reprod. Domest. Anim..

[42] Shen J., Hao Z., Wang J., Hu J., Liu X., Li S., Ke N., Song Y., Lu Y., Hu L. (2021). Comparative Transcriptome Profile Analysis of Longissimus dorsi Muscle Tissues From Two Goat Breeds with Different Meat Production Performance Using RNA-Seq. Front. Genet..

[43] Tai C.S., Chen Y.Y., Chen W.L. (2016). *β*-Lactoglobulin Influences Human Immunity and Promotes Cell Proliferation. BioMed Res. Int..

[44] Liu J., Xu L., Ding X., Ma Y. (2023). Genome-Wide Association Analysis of Reproductive Traits in Chinese Holstein Cattle. Genes.

[45] Sun X., Niu Q., Jiang J., Wang G., Zhou P., Li J., Chen C., Liu L., Xu L., Ren H. (2023). Identifying Candidate Genes for Litter Size and Three Morphological Traits in Youzhou Dark Goats Based on Genome-Wide SNP Markers. Genes.

[46] Verardo L.L., E Silva F.F., Machado M.A., Do Carmo Panetto J.C., De Lima Reis Faza D.R., Otto P.I., Regitano L.C.d.A., da Silva L.O.C., Egito A.A.D., Albuquerque M.D.S.M. (2021). Genome-Wide Analyses Reveal the Genetic Architecture and Candidate Genes of Indicine, Taurine, Synthetic Crossbreds, and Locally Adapted Cattle in Brazil. Front. Genet..

[47] Zhai B., Liu H., Li X., Dai L., Gao Y., Li C., Zhang L., Ding Y., Yu X., Zhang J. (2013). BMP15 Prevents Cumulus Cell Apoptosis Through CCL2 and FBN1 in Porcine Ovaries. Cell. Physiol. Biochem..

[48] Xu S.-S., Gao L., Xie X.-L., Ren Y.-L., Shen Z.-Q., Wang F., Zhang L., Ding Y., Yu X., Zhang J. (2018). Genome-Wide Association Analyses Highlight the Potential for Different Genetic Mechanisms for Litter Size Among Sheep Breeds. Front. Genet..

[49] Padilla J., Jenkins N.T., Thorne P.K., Martin J.S., Rector R.S., Davis J.W., Laughlin M.H. (2014). Transcriptome-wide RNA sequencing analysis of rat skeletal muscle feed arteries. II. Impact of exercise training in obesity. J. Appl. Physiol..

[50] Reyer H., Abou-Soliman I., Schulze M., Henne H., Reinsch N., Schoen J., Wimmers K. (2024). Genome-Wide Association Analysis of Semen Characteristics in Piétrain Boars. Genes.

[51] Wang S., Liu Y., Li S., Chen Y., Liu Y., Yan J., Wu J., Li J., Wang L., Xiang R. (2023). COMMD3-Mediated Endosomal Trafficking of HER2 Inhibits the Progression of Ovarian Carcinoma. Mol. Cancer Res..

[52] Smitchger J.A., Taylor J.B., Mousel M.R., Schaub D., Thorne J.W., Becker G.M., Murdoch B.M. (2024). Genome-wide associations with longevity and reproductive traits in U.S. rangeland ewes. Front. Genet..

[53] Rosenfeld C.R., Liu X., DeSpain K. (2009). Pregnancy modifies the large conductance Ca^2+^ -activated K^+^ channel and cGMP-dependent signaling pathway in uterine vascular smooth muscle. Am. J. Physiol.-Heart Circ. Physiol..

[54] Meurens F., Whale J., Brownlie R., Dybvig T., Thompson D.R., Gerdts V. (2007). Expression of mucosal chemokines TECK/CCL25 and MEC/CCL28 during fetal development of the ovine mucosal immune system. Immunology.

[55] Oberheide K., Puchkov D., Jentsch T.J. (2017). Loss of the Na^+^/H^+^ exchanger NHE8 causes male infertility in mice by disrupting acrosome formation. J. Biol. Chem..

[56] Fang X., Gu B., Chen M., Sun R., Zhang J., Zhao L., Zhao Y. (2023). Genome-Wide Association Study of the Reproductive Traits of the Dazu Black Goat (*Capra hircus*) Using Whole-Genome Resequencing. Genes.

